# Functional roles and applications of polysaccharides in biomedical and food systems

**DOI:** 10.3389/fbioe.2026.1774298

**Published:** 2026-04-02

**Authors:** A. Atepileva, G. Kudaibergen, T. Krivoruchko, A. Zhulikeyeva, N. Amantay, G. Unysheva, Z. Akhmetkarimova, Y. Alzhanov

**Affiliations:** 1 Orthopedic Traumatologist, National Scientific Center of Traumatology and Orthopedics Named Afteracademician N.D. Batpenov, Astana, Kazakhstan; 2 Toxicology and Pharmacology Laboratory, National Center for Biotechnology, Astana, Kazakhstan; 3 Department of Therapy, Karaganda Medical Intercollege, Karaganda, Kazakhstan

**Keywords:** biopolymer, drug delivery system, polysaccharides, regenerative medicine, tissue engineering

## Abstract

Polysaccharides are renewable biopolymers widely used in biomedical and food systems because of their structural diversity, biodegradability, and biocompatibility. This review summarizes recent progress in the use of major polysaccharides, including alginate, chitosan, gellan gum, heparin, and cellulose, in drug delivery, tissue engineering, and food-related applications. Particular attention is given to structure function relationships, controlled release behavior, bioactivity, and material performance in sustainable packaging and functional food design. Current limitations related to processing, stability, and regulatory translation are also discussed. Overall, polysaccharide-based materials provide versatile platforms for the development of safer medical technologies and sustainable food systems.

## Introduction

Polysaccharides are among the most versatile natural macromolecules and are widely distributed in plants, algae, microorganisms, and animals. Their structural diversity, biocompatibility, and biodegradability have driven extensive research across biomedicine, food science, environmental engineering, and sustainable materials development. The global transition toward sustainable and resource-efficient technologies has further intensified interest in polysaccharide-based materials across healthcare, food, and environmental applications (Xue et al., 2025; Benalaya et al., 2024).

In healthcare, polysaccharides are активно studied for tissue engineering, wound healing, and drug delivery because of their hydrogel-forming ability, biological compatibility, and capacity for controlled therapeutic release. In food systems, they play essential techno-functional roles as thickeners, gelling agents, stabilizers, dietary fibers, and bioactive compounds that influence gut microbiota and immune responses. More recently, polysaccharide-derived films and composites have emerged as promising materials for sustainable food packaging, offering biodegradable alternatives to conventional plastics together with active and intelligent packaging functions (Jabeen and Atif, 2024; Farasati Far et al., 2022; Cui et al., 2022; Yang and Wang, 2023; Wu X. et al., 2023; Sepe et al., 2025; Khodadadi Yazdi et al., 2024; Liu et al., 2024). Despite rapid progress, current knowledge remains fragmented across disciplinary boundaries. Biomedical research emphasizes therapeutic performance and biocompatibility, whereas food science focuses on rheology, texture, and preservation. Materials science studies primarily address mechanical strength and barrier behavior. As a result, integrated understanding of structure-function relationships governing polysaccharide performance across healthcare and food technologies is still limited. Comparative evaluation of mechanisms, functional roles, and translational barriers is particularly insufficient in existing reviews (Yu et al., 2026; Azeredo, 2009; Gao et al., 2024).

Another unresolved issue concerns the balance between traditional polysaccharide systems and emerging multifunctional composites. Classical materials such as starch, alginate, and gellan gum continue to dominate applied research. At the same time, reinforced and hybrid systems including nanocellulose-based composites and bioactive polysaccharide matrices with antimicrobial or immunomodulatory activity are rapidly expanding. However, links between molecular architecture, processing strategy, and real-world technological performance remain poorly synthesized (Arshad et al., 2025; Sarfraz et al., 2021; Marasinghe et al., 2024).

Therefore, this review provides a cross-disciplinary synthesis of polysaccharide applications in emerging healthcare and food-related technologies. Particular emphasis is placed on structure-function relationships, underlying physicochemical mechanisms, comparative analysis of major polysaccharide systems, and current limitations affecting scalability, regulation, and industrial translation. By integrating perspectives from biomedicine, food science, and sustainable materials research, this work aims to clarify the technological potential of polysaccharides and outline future research directions for environmentally responsible and health-oriented applications (Baranwal et al., 2022; Chen et al., 2021).

## Healthcare applications

Advanced biomaterials and nanoscale delivery systems are increasingly incorporated into modern healthcare to improve diagnosis, treatment, and long-term disease management. Among these materials, polysaccharides are of particular interest because they combine biocompatibility, biodegradability, and the ability to support tissue regeneration while limiting adverse immune responses. In addition to their structural and carrier functions, certain polysaccharides exhibit intrinsic immunomodulatory activity. *β*-Glucans and chitosan derivatives demonstrate immunomodulatory effects through receptor-mediated pathways, contributing to immune regulation in biomedical applications (Goodridge et al., 2009). *β*-Glucans have been shown to interact with pattern-recognition receptors, including Dectin-1 and Toll-like receptors, leading to macrophage activation and cytokine regulation (Zhao et al., 2025; Iruoghene et al., 2025; Ya et al., 2025). Chitosan derivatives have also been reported to modulate inflammatory signaling and enhance immune cell recruitment during tissue repair (Ya et al., 2025). Drug delivery platforms based on nanocapsules, microparticles, and hydrogels enable localized and controlled release of therapeutic agents, which can enhance efficacy and reduce systematic side effects. Polysaccharide-derived materials are also widely studied in regenerative medicine, where injectable hydrogels, alginate wound dressings, and composite scaffolds are used to support the repair of chronic wounds, burns, and postoperative tissue damage. At the same time, the integration of wearable sensors and implantable monitoring devices allows continuous assessment of physiological parameters and facilitates adaptive treatment strategies. Current research therefore focuses on improving material stability, biological performance, and clinical translation, with the broader goal of expanding the role of polysaccharide-based technologies in personalized and regenerative medicine.

### Tissue engineering

Tissue engineering is an interdisciplinary field aimed at the regeneration or replacement of damaged tissues such as skin, bones, and blood vessels. This approach relies on the cultivation of cells within scaffolds that reproduce key biological and mechanical features of the native extracellular matrix. Effective scaffold design requires a porous architecture, controlled degradation synchronized with tissue formation, structural stability that preserves pore connectivity, and mechanical properties that support cell growth. In addition, scaffold must provide high biocompatibility to limit inflammation reactions while promoting favorable cell–material interactions. Owing to their biodegradability, biocompatibility, and structural tunability, polysaccharides are widely investigated as scaffold materials for tissue engineering applications (LogithKumar et al., 2016; Li et al., 2017; Annabi et al., 2014; Van Vlierberghe et al., 2011; Li R. et al., 2024).

Hydrogels are widely explored as biomimetic matrices for bone regeneration and autologous bone repair because they provide a hydrated three-dimensional environment that supports tissue integration and limits early bacterial colonization. Osteogenic growth factors, such as bone morphogenetic protein (BMP), fibroblast growth factor (FGF), and transforming growth factor-beta (TGF-β) are central regulators of bone cell differentiation and matrix formation (Maisani et al., 2017).

Jung et al. (2019) developed chitosan-based hydrogels incorporating BMP-2 and vancomycin. *In vivo* studies showed sustained antibiotic release that suppressed *Staphylococcus aureus* infection while prolonged BMP-2 exposure promoted bone regeneration and prevented bone mass loss. Such multifunctional hydrogels therefore represent potential therapeutic platforms for osteomyelitis, where infection control and tissue regeneration must occur simultaneously. Related composite systems combining chitosan with silver particles have also been shown to enhance mesenchymal stem cells differentiation from bone marrow during cross-linking, supporting their use as scaffold materials for bone tissue regeneration. Together, these studies demonstrate how antibacterial activity, growth-factor delivery, and stem-cell regulation can be integrated within polysaccharide hydrogels to improve bone repair outcomes.

Injectable polysaccharide-based hydrogels are increasingly used for the restoration of bone, joint, and cartilage tissues because they enable minimally invasive delivery and conformal filling of irregular defects. Several formulations have reached clinical application in Europe and the United States, including viscosupplementation products for osteoarthritis treatment and injectable scaffolds designed to support joint regeneration (Zheng et al., 2022; Xue et al., 2021; Russu et al., 2021; Cevik and Dikici, 2024). Compared with conventional preformed scaffolds, injectable hydrogels allow *in situ* gelation, improved defect coverage, and enhanced patient tolerance.

Chronic wounds and periodontal lesions further illustrate the therapeutic relevance of antimicrobial polysaccharide hydrogels. Such conditions are often associated with persistent colonization by gram-positive and gram-negative bacteria, requiring localized delivery of antimicrobial agents. Polysaccharide-based injectable dressings incorporating compounds such as nanosilver, curcumin, vancomycin, or ornidazole therefore provide controlled drug release while maintaining a moist environment that supports tissue repair. For example, (Zhang et al., 2020), reported a chitosan hydrogel loaded with ornidazole that promoted healing of periodontal Class III furcation defects. Together, these studies indicate that injectable polysaccharide hydrogels combine structural support, antimicrobial protection, and minimally invasive administration, making them versatile platforms for musculoskeletal and wound-healing applications.

Diabetic wounds represent a major clinical challenge because chronic hyperglycemia impairs inflammatory regulation, angiogenesis, and tissue remodeling, thereby increasing susceptibility to infection and delaying repair (Falanga, 2005; Bus and Ph, 2017). Curcumin-loaded chitosan nanoparticles incorporated into collagen-alginate scaffolds significantly improved wound contraction and collagen deposition in streptozotocin-induced diabetic wound models (Venkata et al., 2016). Injectable curcumin-loaded chitosan/oxidized-alginate hydrogels enhanced wound closure and improved histological organization *in vivo*, indicating support for re-epithelialization and tissue regeneration (Li et al., 2012). Commercial polysaccharide-based dressings such as KytoCel®, and Axiostat® have demonstrated clinical utility in wound management and bleeding control (Mason and Clarke, 2015; Perri et al., 2025). Overall, polysaccharide hydrogels represent promising platforms for infection management and tissue repair, although further long-term clinical evaluation remains necessary.

### Healing of skin and tissues

Skin wound healing remains a major clinical challenge and continues to drive the development of advanced injectable hydrogel dressings with antimicrobial, antioxidant, adhesive, and electrically conductive properties. Zhao et al. (2015), Zhao et al. (2017), reported injectable quaternary-ammonium chitosan hydrogels containing polyaniline that exhibit self-healing behavior, strong tissue adhesion, antimicrobial activity, and tunable electrical conductivity. These conductive hydrogels supported regeneration of electrically responsive tissues, including cardiac and neural structures, and promoted full-thickness skin repair *in vivo*.

Wound repair involves coordinated interactions between cells, cytokines, and extracellular matrix remodeling. Recent studies therefore focus on polysaccharide-based hybrid hydrogels incorporating bioactive components such as growth factors (EGF, TGF-*β*, VEGF.), graphene oxide, and mesenchymal stem cells, which enhance granulation tissue formation, collagen deposition, and angiogenesis during healing (Zhang et al., 2020; Chen et al., 2019; Chen et al., 2018; Tian et al., 2021; Zang et al., 2019). Despite these advances, only a limited number of injectable polysaccharide hydrogels have reached clinical translation. Commercial alginate-based dressings (Purilon Gel® and Saf-gel®), support moist wound environments and burn healing, whereas hyaluronic-acid hydrogels (Restylane®) serve as injectable scaffolds for dental pulp regeneration and promote angiogenesis through controlled degradation (Barbu et al., 2021; Chrepa et al., 2017).

### Drug delivery

The concept of targeted drug delivery, often referred to as the “Magic bullet,” was first introduced by the notable German scientist Paul Ehrlich in 1906. In his vision, the “magic bullet” represents an ideal medication with the ability to independently identify the source of an illness or the focal point of a disease, effectively targeting and addressing them while sparing healthy organs and tissues from impact.

**FIGURE 1:**
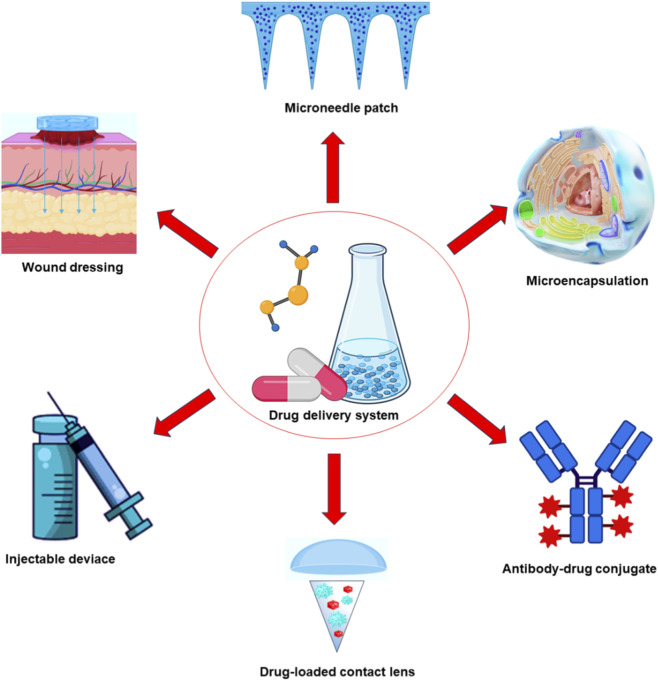
Examples of different drug delivery approaches.

Drug Delivery Systems (DDS) are designed to transport therapeutic agents to specific biological targets in a controlled and efficient manner (Alharbi et al., 2024; Kandile et al., 2024). These platforms improve treatment efficacy by enabling sustained release, spatial targeting, and reduced systemic toxicity. As illustrated in Figure 1, polysaccharide-based DDS support diverse biomedical formats, including microneedle patches, wound dressings, injectable carriers, microencapsulation systems, antibody-drug conjugates, and drug-loaded contact lenses. Recent research therefore focuses on intelligent and stimuli-responsive delivery strategies that allow precise control over dose, timing, and localization of drug action. Natural polysaccharides are particularly attractive for these applications because of their biocompatibility, biodegradability, and structural tenability (Luo and Wang, 2014; Alvarez-Lorenzo et al., 2013; Ahsan et al., 2018; Ali and Ahmed, 2018; Hamedi et al., 2018; Lee et al., 2021).

Chitosan is widely used in drug delivery because of its reactive hydroxyl and amino groups, biocompatibility, biodegradability, and intrinsic antibacterial activity (Shariatinia, 2019; Muanprasat and Chatsudthipong, 2017; Safari et al., 2015). Ryu et al. developed a waterproof catechol-grafted chitosan adhesive (IP plaster) produced by lyophilization, which enables effective sealing of intestinal neoanastomosis sites and reduces the risk of postoperative anastomotic failure after colorectal surgery (Tripodo et al., 2015; Luo et al., 2019). The strong tissue adhesion of this material also allows its use as a localized drug reservoir, and therapeutic efficacy has been demonstrated in models of peritoneal tumor dissemination. These results illustrate how chemical modification and adhesive functionality expand the role of chitosan from a conventional carrier to a multifunctional platform for localized and controlled drug delivery in complex intra-abdominal conditions.

**FIGURE 2:**
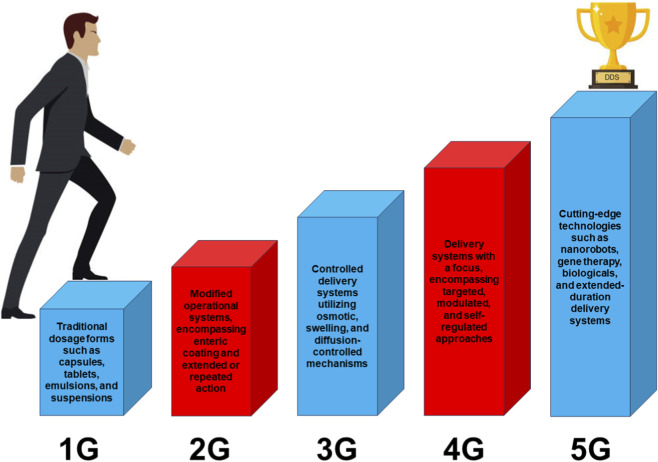
Evolution of drug delivery systems from traditional dosage forms to advanced targeted and stimuli-responsive platforms (1G-5G).

Rapid progress in DDS has changed both medication administration and therapeutic strategies (Figure 2). As shown in the generational evolution from 1G to 5G platforms, modern DDS increasingly rely on controlled release, targeting, and stimuli-responsive mechanisms. Natural polysaccharides contribute to these advances through their gel-forming ability, chemical tunability, and biocompatibility, enabling applications in nanocarriers, tissue engineering scaffolds, and functional biomedical materials. Recent studies also focus on targeted delivery to resistant deep-seated tumors. For example, hyaluronic acid can bind CD44 receptors on cancer cells and support selective accumulation of drug carriers in tumor tissue (Huang and Huang, 2018). Gao et al. (2019) developed a multifunctional PDA/Hb-based nanocarrier loaded with doxorubicin and nitric-oxide donors and further functionalized with hyaluronic acid, resulting in a biocompatible system with tumor-targeting and controlled-release behavior. Drug release in this platform is regulated by the tumor microenvironment, including enzymatic degradation and local pH conditions. Evolution of drug delivery systems from traditional dosage forms to advanced targeted and stimuli-responsive platforms (1G-5G).

Alginate is widely used for the fabrication of drug delivery carriers because of its biocompatibility, mild gelation conditions, and structural tenability (Uyen et al., 2020; Agüero et al., 2017). Wu et al. developed pH-responsive alginate hydrogel beads containing chlorpyrifos loaded into polydopamine-modified attapulgite through electrostatic and hydrogen-bond interactions (Wu et al., 2021). These beads enabled controlled release under alkaline conditions, improved resistance to UV-induced degradation, and maintained favorable biocompatibility, demonstrating their suitability as delivery platforms. Related strategies have also been reported for antibiotic loading. Xue and co-workers prepared dopamine-modified alginate hydrogels with enhanced adsorption capacity for gatifloxacin and improved structural stability compared with unmodified alginate systems (Xue et al., 2021). Together, these studies illustrate how chemical modification and composite design expand the controlled-release performance and functional stability of alginate-based delivery materials.

Tаng and co-workers (Tang et al., 2017) reported a pH-responsive nanocapsule system based on dopamine-conjugated alginate coordinated with Fe^3+^ ions and deposited onto doxorubicin-loaded ZIF-8 nanoparticles (Shieh et al., 2015). The resulting nanocapsules showed high drug loading capacity and pronounced cytotoxic activity toward cancer cells, supporting their potential use in targeted delivery. More broadly, hydrogel-based polysaccharide matrices provide hydrated three-dimensional networks that enable efficient encapsulation of water-soluble small molecules and macromolecular therapeutics (Bhatt et al., 2010; Park et al., 2010). These findings highlight how metal coordination, polymer modification, and porous carrier integration can be combined to achieve pH-controlled and structurally stable drug delivery platforms.

## Food application

In food systems, polysaccharides function not only as structural biopolymers but as multifunctional ingredients that regulate rheology, texture, physical stability, and biological activity. Their technological significance is governed by hydration-driven molecular organization and polymer-water interactions, which determine viscosity development, gel formation, and structural integrity across diverse food matrices Consequently, the functional roles of food-related polysaccharides can be categorized into rheology control and thickening, gelation and structuring, stabilization of emulsions and dispersions, dietary fiber functionality, and bioactivity-associated health effects (Yang et al., 2020; Nishinari et al., 2000; Dickinson, 2009). Beyond their classical techno-functional roles, increasing attention has been directed toward sustainability-oriented applications of polysaccharides in edible films and biodegradable packaging materials. Unlike petroleum-derived plastics, polysaccharide-based materials offer renewability, biodegradability, and the possibility of incorporating active or intelligent functions that contribute to food preservation and safety (Sarfraz et al., 2021; Aaliya et al., 2023; Rostamabadi et al., 2023; Cazón et al., 2017). This convergence between structural functionality in foods and material performance in packaging underscores the expanding cross-disciplinary relevance of polysaccharides in contemporary food science (dos Santos et al., 2016).

### Functional roles of polysaccharides in food systems

At the mechanistic level, viscosity enhancement arises primarily from chain entanglement, molecular weight distribution, and shear-dependent flow behavior, while ionically or thermally induced gelation generates three-dimensional hydrogel networks responsible for mechanical strength and texture perception (Nishinari et al., 2000; Cazón et al., 2017). Interfacial adsorption and steric stabilization further enable the formation of stable emulsions and dispersions (Dickinson, 2009). In parallel, specific dietary polysaccharides such as pectins, hemicelluloses, and *β*-glucans undergo microbial fermentation in the gastrointestinal tract, producing short-chain fatty acids and modulating metabolic, inflammatory, and immune pathways (Host, 2018). Certain polysaccharides, including *β*-glucans and chitosan derivatives, also exhibit antimicrobial properties relevant to food preservation and postharvest protection. These observations show that the functions of food polysaccharides are determined by clear relationships between molecular structure, physicochemical behavior, and biological activity. The main functional categories and their underlying mechanisms are summarized in Table 1.

**TABLE 1:** Functional roles and molecular mechanisms of major polysaccharides in food systems.

No	Category	Major polysaccharides	Core mechanisms	Refs
1	General functional roles in food systems	Multiple hydrocolloids	Hydration-driven structuring; polymer-water interactions; rheology-molecular weight relationships; hydrogel network formation	Gao et al. (2024), Mechanisms et al. (2023), Alam et al. (2025)
2	Thickening and rheology control	Guar gum, xanthan gum, pectin	Chain entanglement; high viscosity at low concentration; shear-thinning flow sensory tribology	Saha and Bhattacharya (2010), Tahmouzi et al. (2023), Theocharidou et al. (2021), Xu et al. (2025), Zhong et al. (2024)
3	Gelling and structuring systems	Alginate, gellan gum	Ca^2+^-mediated ionic crosslinking; egg-box architecture; thermoreversible gelation; mechanical stability	Yu et al. (2026), Cao et al. (2024); Gomes et al. (2023), Gadziński et al. (2023), Marques et al. (2025)
4	Stabilization of emulsions and dispersions	Pectin, mixed hydrocolloids	Interfacial adsorption; steric stabilization; viscosity-controlled droplet mobility	Mechanisms et al. (2023), Jia et al. (2024), Manzoor et al. (2022)
5	Multifunctional structuring polysaccharide and fermentable dietary fiber	High- and low-methoxyl pectin	Degree-of-esterification-dependent gelation; Ca^2+^ crosslinking; pH sensitivity; microbial fermentability and SCFA generation	Jia et al. (2024), Xiang et al. (2024), Nath et al. (2023), Sharma et al. (2026), Nohemi et al., 2025; Paz (2025)
6	High-viscosity thickener with synergistic rheological behavior	Guar gum; guar-xanthan systems	Rapid hydration of galactomannan backbone; synergistic viscosity enhancement; texture and mouthfeel modulation	Tahmouzi et al. (2023), Xu et al. (2025), Mudgil et al. (2014)
7	Dietary polysaccharides and gut microbiota modulation	Hemicellulose, pectin, *β*-glucans	Microbial fermentation; short-chain fatty acid production; intestinal barrier regulation; metabolic and inflammatory modulation	Zhao et al. (2025), Iruoghene et al. (2025), Ana, 2022; Sun et al. (2025), Zhang et al. (2024), Shoukat and Sorrentino (2021)
8	Antimicrobial, film-forming polysaccharide	Chitosan and derivatives	Cationic membrane interaction; antimicrobial disruption; bioactive coating formation; postharvest protection	Kumar and Tyagi (2025), Dai et al. (2025), Wang et al. (2024)
9	Edible films and sustainable packaging	Alginate, nanocellulose, gellan gum, chitosan	Biodegradable film formation; moisture sensitivity; mechanical reinforcement *via* composites	Fan et al. (2025), Li et al. (2024b)

Overall, the functional roles of food-related polysaccharides follow clear structure-property-function relationships. These relationships connect physicochemical structuring with gastrointestinal bioactivity and material performance. As shown in Table 1, hydration-driven rheology, ionic gelation, interfacial stabilization, and microbial fermentation determine both technological behavior and health-related effects in food systems. The growing use of polysaccharides in biodegradable and active packaging further demonstrates the transition of traditional hydrocolloids beyond formulation toward sustainability-oriented applications. Future progress will require improved molecular characterization, standardized processing strategies, and clearer regulatory translation to enable reliable industrial implementation. This growing interest in sustainable polysaccharide materials is closely linked to the limitations of conventional petroleum-based plastics used in food packaging.

### Polysaccharide-based biodegradable and active packaging

**FIGURE 3:**
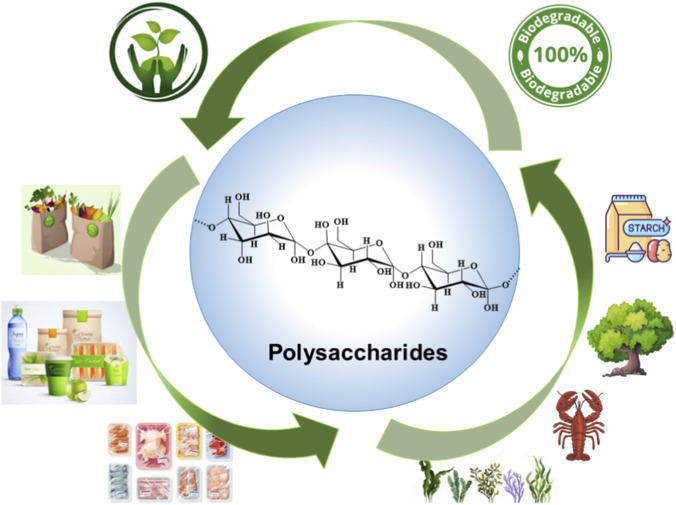
Polysaccharide-based biodegradable coatings and films for food packaging applications.

During the initial years of the 20th century, Leo H. Baekeland introduced the first synthetic plastic material, marking the onset of the plastic era. This transformative development has significantly impacted human life, with plastic-based materials now integral to various aspects of daily living. Presently, these materials are essential components of food packaging, effectively extending the shelf life of food products due to their exceptional preservation capabilities. In the past few years, increased awareness of the drawbacks of petroleum-derived products has spurred demand for environmentally friendly alternatives to conventional plastic packaging materials (Aaliya et al., 2023; Rostamabadi et al., 2023). In response, there has been growing interest in utilizing nature-inspired polymers for manufacturing food packaging materials (Figure 3.). The utilization of diverse biopolymers, including proteins, lipids, and polysaccharides, has enabled the development of biodegradable packaging materials. Among these, polysaccharides stand out due to their biodegradability, non-toxicity, flexibility, accessibility, and abundance. This focuses on recent innovations in polysaccharide-based food packaging materials, highlighting their importance in promoting a healthier lifestyle and a safer environment (Rostamabadi et al., 2023).

### Starch-based biodegradable films and composites

Starch, a key plant carbohydrate, is produced by organelles and stored in amyloplasts. Varieties like corn, potato, wheat, rice, and cassava offer distinct properties, from physicochemical to thermal. Through chemical, physical, or enzymatic modifications, native starches are tailored to enhance functionality. Abundant and cost-effective, starch is widely used in food packaging, either alone or with reinforcing agents. Typically containing 15%–30% amylose, starch’s linear structure comprises *α*-1,4-linked glucose units, while amylopectin’s branched form includes *α*-1,6 linkages and side chains. These structural variations affect starch’s properties like gelatinization and water absorption. With adaptability for various packaging needs, starch undergoes modifications for active or intelligent packaging, making it biodegradable and compostable. However, its susceptibility to moisture can lead to disintegration, providing a favorable environment for microbial degradation under specific conditions (Aaliya et al., 2022).

Despite its natural degradability and compostability, the high moisture sensitivity of starch restricts its standalone use in packaging. To overcome this, starch is often chemically, enzymatically, or physically modified to enhance its mechanical and barrier properties. For instance, films reinforced with cellulose nanocrystals exhibit improved elongation and resistance to moisture. Basiak et al. (2017) investigated the impact of starch origin (wheat, corn, and potato) on film characteristics. Their results showed that the amylose-to-amylopectin ratio strongly governs viscosity, drying shrinkage, final thickness, and hydration behavior. Films derived from potato starch, which is rich in amylopectin, exhibited lower water solubility and reduced oxygen permeability. Colussi et al. (2017) reported complementary findings for rice-starch films. Moderate amylose content resulted in higher tensile strength, highlighting the importance of molecular composition in determining mechanical performance. However, systematic comparisons of crystallinity patterns and their relationship to film properties remain limited. To enhance functionality, starch matrices are frequently combined with reinforcing or bioactive components, including chitosan (Fonseca-García et al., 2021), cellulose (Zhang et al., 2023), plant oils (Gao et al., 2020), proteins (Xu et al., 2023), nanocomposites (Wu H. et al., 2023), cellulose nanocrystals (Ren et al., 2023), and agricultural by-products (Bhat et al., 2013). These additives form composite structures with improved mechanical integrity, barrier performance, and preservation capacity. Benito-González et al. (2022) demonstrated that rice-based biocomposites produced by melt mixing exhibit increased crystallinity and stiffness. The effect was attributed to flour-derived impurities that promote ordered network formation. Ren et al. (2023) further showed that cellulose nanocrystals obtained from quinoa straw enhance elongation at break and water resistance in corn-starch films, indicating the potential of low-cost lignocellulosic residues as functional reinforcements.

Active packaging strategies further expand the performance of starch-based materials. Zhang et al. (2022) developed composite films containing octenyl-succinate cassava starch, chitosan, nano-ZnO, and *ε*-poly-L-lysine. These films exhibited improved thermal stability and prolonged preservation of cherries. Wang et al. (2022) prepared edible corn-starch films enriched with Coffee arabica and Coffea canephora oils, resulting in enhanced mechanical strength, ultraviolet shielding, and antibacterial activity against *Staphylococcus aureus*. Collectively, these studies demonstrate that rational compositional design and multifunctional reinforcement are essential for overcoming intrinsic limitations of starch and enabling its practical use in sustainable food packaging systems.

### Cellulose and nanocellulose for sustainable food packaging

Cellulose, a polysaccharide with a linear, stereoregular, and semicrystalline structure, consists of glucose units linked by *β*-(1-4) glycosidic linkages and possesses three OH groups. It is widely available in plants, agricultural residues, marine organism shells, and microorganisms (Azeredo et al., 2019). Addressing the sensitivity of cellulose and its derivatives to gaseous and liquid water, along with insufficient interfacial adhesion, poses a significant challenge. Consequently, modifying cellulose fibers chemically or physically can enhance their compatibility, reduce hydrophilicity, and boost thermal stability. This leads to improved dispersion in polymer matrices, enhanced barrier properties, and increased mechanical strength of food packaging materials. Marrez et al. (2019) conducted a study demonstrating that environmentally friendly nanocomposite films, comprising cellulose acetate and nanosilver, exhibited reduced swelling (0.28–0.65) and lower water content (25%–36%). This suggests the lower hydrophilicity of cellulose acetate compared to the more hydrophilic polyvinyl alcohol (PVA). Meanwhile, (Wang et al., 2016) investigated eco-friendly packaging derived entirely from lignocellulose. Their findings revealed that incorporating oxalic acid-modified micro fibrillated cellulose with nanosized alkali lignin yielded significant synergistic effects, notably enhancing the water vapor barrier and mechanical strength of cellulose-based packaging. The commercial appeal of micro- and nanosized cellulose products has grown considerably in recent years due to their biodegradability, making them ideal for sustainable packaging. Additionally, these cellulose-based materials, known as micro fibrillated cellulose or cellulose fibers, contribute to prolonging the shelf life of fruits and vegetables by providing preservative and barrier properties (Liu et al., 2021).

Recent advances in cellulose-based packaging focus on nanocellulose, which offers superior mechanical strength and barrier properties. Incorporating oxalic acid-modified cellulose microfibrils with nanosized lignin has been shown to significantly enhance water vapor resistance and tensile strength, making these materials particularly effective for packaging fresh produce. Similarly, cellulose acetate films with nanosilver have shown antimicrobial properties, making them suitable for extending the shelf life of perishable foods. Additionally, hydrophobic cellulose has been identified as a key component in advanced nanocomposites for dry food packaging (Vanitha and Kavitha, 2021).

Nanocellulose has recently emerged as an important reinforcing component in polysaccharide-based food packaging owing to its high crystallinity, mechanical strength, and low gas permeability. Nanocellulose and its derivatives have been extensively reviewed as sustainable and biodegradable reinforcements that improve the thermo-mechanical properties and barrier performance of biopolymer films for food packaging applications (Zhang et al., 2022; Wang et al., 2022; Marrez et al., 2019; Wang et al., 2016; Liu et al., 2021; Ahankari et al., 2021). When incorporated into starch, alginate, or chitosan matrices, nanocellulose significantly enhances tensile strength, structural cohesion, and resistance to moisture diffusion, thereby addressing several limitations of neat polysaccharide films (Xu et al., 2024). Compared with ionically crosslinked systems such as alginate or gellan, nanocellulose-reinforced composites generally demonstrate superior barrier performance and dimensional stability under humid conditions, as reported in recent reviews (Reddy et al., 2025). Nevertheless, challenges related to nanoparticle dispersion, interfacial compatibility, scalable processing methods, and industrial cost remain critical barriers to commercialization (Fotie et al., 2020).

### Gellan gum–based films and functional coatings

Gellan gum, a complex polysaccharide with a molecular weight of approximately 0.5 × 10^6^ Da, composed of repeating units of *α*-L-rhamnose, *β*-D-glucose, and *β*-D-glucuronate in a fixed 1:2:1 M ratio. Its structural uniqueness lies in the presence of acyl groups attached to specific glucose residues, which influence its hydration and gel-forming capabilities. The behavior of gellan gum in water is governed by the type of ions present; divalent ions, such as calcium, inhibit hydration and require elevated temperatures for solubilization, unlike monovalent ions, which promote easier dissolution. Additionally, external factors like water hardness and the type of gellan gum being used significantly impact its hydration efficiency. When dissolved with sequestrants, GG solutions exhibit high viscosity and are less prone to shear thinning than xanthan gum, although their viscosity decreases at elevated temperatures, simplifying handling in industrial processes. GG forms gels in water through mechanisms such as double-helix bonding and ionic cross-linking. The degree of esterification directly affects the texture of the resulting gels, allowing for customization in various applications. For example, it is frequently used in the production of low-calorie jellies and jams, where it enhances stability in acidic environments, improves clarity, and preserves flavor. Additionally, GG-based coatings are applied to fried foods to improve texture and reduce oil absorption, contributing to higher product quality.

Hernández-García et al. (2021) devised bilayer films using cassava and maize starch, augmented with GG/XG and polyesters. The WVP and OP of Starch-GG blended films diminished after 1 and 5 weeks of storage at 25 °C and 53% RH, as the gum reinforced a sturdier network, curbing mass transfer and decreasing WVP and OP throughout the storage period. Wu et al. (2021) crafted active and intelligent films employing GG, Clitoria ternatea extract, and heat-treated soy protein isolate (HSPI). The tensile strength of GG blend films declined with HSPI incorporation, while percent elongation increased. HSPI disrupted bond formation among GG molecules, elevating elastic modulus and enhancing film flexibility. Conversely, C. ternatea extract composite films exhibited a reverse trend, likely due to anthocyanin-rich extract interference hindering GG-HSPI interactions. Guo et al. (2020) integrated nisin into GG and guar gum (GU) blend films. Native GG film tensile strength increased with GU addition, facilitated by hydrogen bond interaction formation between GG and GU. Percent elongation rose post a 40% GU and nisin addition, owing to intermolecular association among gum molecules and nisin. Nisin addition to GG:GU blend film demonstrated maximum inhibition zones for *Bacillus subtilis* (7.07 mm) and *E. coli* (4.30 mm) at 5 mg/mL concentration, suggesting potential for packaging high-moisture foods and inhibiting microbial decay by reducing relative humidity.

Gellan gum has traditionally been valued in food systems for its gelling, stabilizing, and thickening functions. More recently, it has attracted attention as a biodegradable matrix for sustainable packaging films. This emerging application is closely related to the unique gelation mechanism of gellan, which involves the formation of ordered double-helical structures that aggregate into three-dimensional networks in the presence of cations. Such network organization provides high gel clarity, mechanical rigidity, and tunable barrier performance, thereby stimulating interest in gellan-based packaging materials. Compared with starch-derived matrices, gellan films generally exhibit greater structural homogeneity and reduced sensitivity to retrogradation, although they remain more susceptible to moisture than nanocellulose-reinforced systems. The physicochemical properties of gellan films can be further modulated through ionic strength, polymer concentration, and composite formulation, allowing control over tensile strength, oxygen permeability, and water vapor transport. Despite these advantages, large-scale application is still constrained by brittleness and humidity-induced instability. Consequently, recent studies increasingly focus on composite gellan systems and crosslinking strategies aimed at improving mechanical durability and barrier performance.

### Alginate films and active packaging systems

Alginate, a seaweed-derived anionic polysaccharide, is widely used for the fabrication of biodegradable food packaging films due to its ionically crosslinkable structure and film-forming ability (Roy and Rhim, 2019). Its polymer chains contain mannuronic and guluronic acid residues arranged in homopolymeric and alternating blocks, which govern cation-mediated gelation behavior. In the presence of divalent ions such as Ca^2+^, Zn^2+^, or Mg^2+^, alginate undergoes cooperative crosslinking that produces the well-known “egg-box” network architecture (Giz et al., 2020; Garavand et al., 2017; Satriaji et al., 2020). This ionically crosslinked network provides structural cohesion, moderate mechanical strength, and effective oxygen barrier performance, explaining the broad use of alginate in edible coatings and packaging films. Compared with starch-based matrices, alginate gels generally exhibit lower solubility and improved structural stability, while remaining more sensitive to moisture than nanocellulose-reinforced systems. Relative to gellan gum, alginate forms softer but more flexible networks due to differences in helix aggregation and junction-zone density. Despite these advantages, the high hydrophilicity of alginate limits water-vapor resistance and dimensional stability under humid conditions. Consequently, recent research has focused on composite alginate films, multivalent crosslinking, and nanoparticle reinforcement to enhance mechanical durability and barrier performance in sustainable food packaging applications.

Alginate films are particularly valued for their ability to incorporate active ingredients, making them suitable for functional packaging. However, neat alginate films are inherently brittle and exhibit limited mechanical strength and moisture resistance, which restrict their direct application (Li et al., 2017). To overcome these limitations, formulations are often enhanced with plasticizers, polymers, or bioactive compounds. For example, the addition of antioxidants or antimicrobial agents not only improves the physical properties of the films but also provides protective functionality (Zhao et al., 2025).

Alginate exhibits favorable film-forming ability and is therefore widely explored for bio-based and functional food packaging applications. In addition to providing a structural matrix, alginate networks can act as carriers for bioactive compounds, enabling antioxidant, antimicrobial, and ultraviolet-blocking functions that contribute to shelf-life extension in products such as meat, bread, and chili (Santos et al., 2022; Shankar and Rhim, 2018; Tran et al., 2020; Rukmanikrishnan et al., 2020; Riahi et al., 2022). Alginate films have also been applied in intelligent packaging systems, including color-changing freshness indicators for real-time monitoring of perishable foods such as fish, milk, and meat (Liu et al., 2019; Zheng et al., 2023). However, neat alginate films remain brittle and exhibit limited mechanical strength, barrier performance, and hydrophobicity, largely due to their intrinsic hydrophilicity (Santos et al., 2022). To overcome these limitations, composite strategies involving plasticizers, crosslinkers, secondary polymers, and functional additives are widely employed, leading to improved structural stability and multifunctional activity (Zhang et al., 2022; Shankar and Rhim, 2018; Tran et al., 2020). Despite these advances, challenges related to moisture sensitivity, processability, production cost, and the absence of standardized large-scale extraction technologies for seaweed-derived polymers continue to hinder industrial translation.

## Conclusion

Polysaccharides are widely used in biomedical and food systems because of their biodegradability, biocompatibility, and structural versatility. Their ability to control hydration, bioactivity, and material organization supports applications in drug delivery, tissue engineering, and functional food design. However, several limitations still restrict their broader translation. These include incomplete understanding of structure–function relationships during processing, variability in degradation behavior, and the need for scalable and standardized manufacturing strategies. In addition, regulatory approval remains a major challenge, particularly for biomedical materials requiring safety validation, long-term biocompatibility assessment, and compliance with food and medical regulatory frameworks. Future research should therefore focus on molecular-level characterization, reproducible processing approaches, and clearer regulatory pathways that support safe and reliable industrial implementation. Addressing these issues will enable wider integration of polysaccharide-based materials into medical technologies and sustainable food systems.
